# Supplementary material for: Modelling Skylarks (Alauda arvensis) to Predict Impacts of Changes in Land Management and Policy: Development and Testing of an Agent-Based Model
Source: PLoS One. 2013 Jun 6;8(6):e65803. doi: 10.1371/journal.pone.0065803 (PMC3675089; doi:10.1371/journal.pone.0065803)
Supplement: Supporting Information S4 — The skylark ODdox as a zipped archive. (ZIP) [file pone.0065803.s004.zip › Skylark_ODdox/class_raster_map-members.html]

ALMaSS Skylark ODdox: Member List


ALMaSS Skylark ODdox
 2.0


- Main Page
- Related Pages
- Classes
- Files

- Class List
- Class Index
- Class Hierarchy
- Class Members

RasterMap Member List

This is the complete list of members for RasterMap, including all inherited members.

|  |  |  |
| --- | --- | --- |
| Get(int a\_x, int a\_y) | RasterMap | inline |
| GetID(void) | RasterMap | inline |
| GetMagicP(int a\_x, int a\_y) | RasterMap | inline |
| m\_height | RasterMap | private |
| m\_id | RasterMap | private |
| m\_map | RasterMap | private |
| m\_width | RasterMap | private |
| m\_x | RasterMap | private |
| m\_y | RasterMap | private |
| Manipulation1() | RasterMap |  |
| MapHeight(void) | RasterMap | inline |
| MapWidth(void) | RasterMap | inline |
| Put(int a\_x, int a\_y, int a\_elem) | RasterMap | inline |
| RasterMap(const char \*a\_mapfile) | RasterMap |  |
| ~RasterMap(void) | RasterMap |  |


- Generated on Thu Jan 10 2013 13:15:37 for ALMaSS Skylark ODdox by
   1.8.1.1
